# Dynamics of intracellular stress-induced tRNA trafficking

**DOI:** 10.1093/nar/gky1208

**Published:** 2018-11-28

**Authors:** Rabin Dhakal, Chunyi Tong, Sean Anderson, Anna S Kashina, Barry Cooperman, Haim H Bau

**Affiliations:** 1 Department of Mechanical Engineering and Applied mechanics, University of Pennsylvania, Philadelphia, PA 19104, USA; 2 Department of Biomedical Sciences, School of Veterinary Medicine, University of Pennsylvania, Philadelphia, PA 19104, USA; 3 Department of Chemistry, University of Pennsylvania, Philadelphia, PA 19014, USA

## Abstract

Stress is known to induce retrograde tRNA translocation from the cytoplasm to the nucleus but translocation kinetics and tRNA-spatial distribution have not been characterized previously. We microinject fluorescently-labeled tRNA into living cells and use confocal microscopy to image tRNA spatial distribution in single cells at various levels of starvation and to determine translocation rate constants. Retrograde tRNA translocation occurs reversibly, within minutes after nutrition depletion of the extracellular medium. Such nutritional starvation leads to down-regulation of tRNA nuclear import and nearly complete curtailment of its nuclear export. Nuclear tRNA accumulation is suppressed in cells treated with the translation inhibitor puromycin, but is enhanced in cells treated with the microtubule inhibitor nocodazole. tRNA in the cytoplasm exhibits distinct spatial distribution inconsistent with diffusion, implying that such distribution is actively maintained. We propose that tRNA biological complexes and/or cytoplasmic electric fields are the likely regulators of cytoplasmic tRNA spatial distribution.

## INTRODUCTION

Transfer ribonucleic acid (tRNA) is a 76–93 nucleotide-long adaptor molecule that links a given amino acid to a specific messenger RNA (mRNA) codon and transfers this amino acid to a nascent polypeptide chain at the ribosomal site of protein synthesis (1). Until recently, it was believed that mature tRNA molecules, following their transcription in the nucleus, are exported to the cytoplasm to undergo aminoacylation and participate in translation (2). Only recently has it been recognized that mature tRNA undergoes retrotranslocation from the cytosol into the nucleus, a reversible process having important roles in cell biology (3). This phenomenon has been most extensively studied in *Saccharomyces cerevisiae* (2,4–7). In addition to conventional splicing in the nucleus, followed by aminoacylation and translation in the cytoplasm, tRNAs also undergo some splicing in the cytoplasm (8), posttranscriptional modification in both the nucleus and the cytoplasm (4), and minor aminoacylation in the nucleus (9), where retrotranslocation functions as a quality control mechanism to repair or degrade aberrant tRNA (4,6,7,10). Retro-translocation of tRNA from the cytosol into the nucleus has been demonstrated under stress conditions such as nutrient deprivation (2,6,7,10–15) and heat treatment (12,13) in both *S. cerevisiae* and mammalian cells (3). tRNA nuclear import has also been implicated in assisting viral (e.g. HIV-1) transport (16,17). Translation in the nucleus has also been suggested (18), but this suggestion remains controversial (19).

Defects in and deregulation of tRNA expression are linked to a number of diseases and disorders, including cancer and neuro-degeneration (20,21). Cancer cells exhibit elevated tRNA levels (20), and tRNA binding to cytochrome C (22–25), resulting in tRNA-mediated inhibition of cytochrome C in mitochondria, is implicated in the shift of cancer cells to anaerobic respiration (26,27).

Despite its emerging role in multiple biological processes, little is known about the subcellular distribution of different tRNA pools and its dynamics under physiological changes, e.g., during stress response. A better understanding of tRNA nuclear/cytoplasmic trafficking mechanisms will aid in elucidating how cells regulate tRNA subcellular distribution, what factors affect tRNA trafficking, how tRNA trafficking differs between healthy and diseased/cancerous cells, and how viruses such as HIV take advantage of tRNA trafficking mechanisms to enter the nucleus.

Prior studies of tRNA trafficking have mostly relied on fluorescent *in-situ* hybridization (FISH) (28) that provides static snapshots of tRNA distribution in fixed cells, but no real-time information on tRNA dynamics. Barhoom *et al.* (29,30) previously have demonstrated that rhodamine labeled tRNA (rhd-tRNA) can be aminoacylated within cells and participate in protein synthesis. Here, we microinject rhd-tRNA into cultured mouse embryonic fibroblasts (MEFs) and image intracellular tRNA dynamics in live cells.

Our results demonstrate that, during nutrient deprivation, tRNA rapidly retro-translocates from the cytoplasm into the nucleus. In agreement with earlier studies (11), this transport is both actively regulated and reversible. Since all the rhd-tRNA originates in the cytoplasm, no nuclear tRNA synthesis is required to support the reversible tRNA retrotranslocation that we observe. Retrograde nuclear tRNA accumulation is suppressed in cells treated with the translation inhibitor puromycin and is enhanced in cells treated with the microtubule inhibitor nocodazole. Strikingly, tRNA exhibits a distinct, non-linear distribution in the cytoplasm that is inconsistent with molecular diffusion. Higher tRNA concentration is maintained in the vicinity of the nuclear envelope, possibly at the main sites of protein synthesis. We formulate a simple lumped parameter model to estimate the rate constants for tRNA nuclear import and export as functions of stress level, and examine various causative mechanisms for the unexpected tRNA concentration distribution in the cytoplasm. Our work constitutes the first quantitative analysis of tRNA dynamics in a eukaryotic cell.

## MATERIALS AND METHODS

### Fluorescently labeled tRNA

Rhodamine-labeled bulk tRNA (bulk rhd-tRNA) was prepared as previously described (29).

### Cell culture

Spontaneously immortalized Mouse Embryonic Fibroblasts (MEF) were cultured in 44.5% DMEM (HyClone), 44.5% F10 nutrient mix, 10% fetal bovine serum (HyClone), and 1% Penicillin/Streptomycin mix on glass-bottom cell culture dishes (MatTek Corp.) and kept in a Fisher Isotemp incubator at 37°C and 5% CO_2_. For nutritional stress, culture media was mixed with PBS at various proportions. Pure PBS solution corresponded to complete nutritional starvation (0% nutrition).

### Microinjection

**Figure 1.**
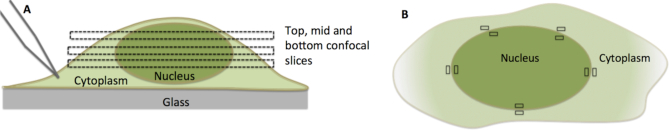
Cartoon of a Mouse Embryonic Fibroblast (MEF). (**A**) Cell cross-section and confocal slices at cell's bottom, mid-height, and top. (**B**) Mid plane cross-section. The average fluorescence intensity emitted from the indicated 5 × 5 pixel rectangular regions next to the nuclear membrane was used to determine the ratio between the nuclear and cytoplasmic fluorescence intensity (FIR) and in the model we use to estimate reaction rate constants.

Bulk rhd-tRNA was dissolved in molecular grade water and back loaded into filamented, pulled quartz micropipettes (S9). The rhd-tRNA solution was ∼25 μM, enabling prolonged observations while maintaining linearity between rhd-tRNA concentration and emission intensity. The loaded micropipettes were then connected to an Eppendorf Femtojet microinjection system and mounted on an Eppendorf Transferman NK2 piezoelectric micromanipulator attached to the Leica DMI4000 Microscope with Yokagawa CSU-X1 Spinning Disk Confocal Attachment. Cells were microinjected with rhd-tRNA volume equivalent to ∼10% of the cell volume (to an average cellular concentration of ∼2.3 μM rhd-tRNA) at a point away from the nucleus (Figure 1). Similar procedures were used when cells were injected with 25 μM FAM-80-DNA in place of rhd-tRNA. Cell cultures were maintained in a stage incubator (LCI Chamlide) at 37°C and 5% CO_2_.

### Imaging

Spinning disk confocal images were collected at the cell's bottom, mid-height, and top (Figure 1A) and digitized with Matlab™ by extracting the intensity of the green color. To estimate reaction rate constants, cytoplasmic intensity was determined by averaging emission intensity from the indicated 5 pixel × 5 pixel rectangular regions next to the nuclear membrane (Figure 1B) and from the spatially-averaged cytoplasmic intensity.

## RESULTS

### tRNA retrograde translocation under starvation conditions

**Figure 2.**
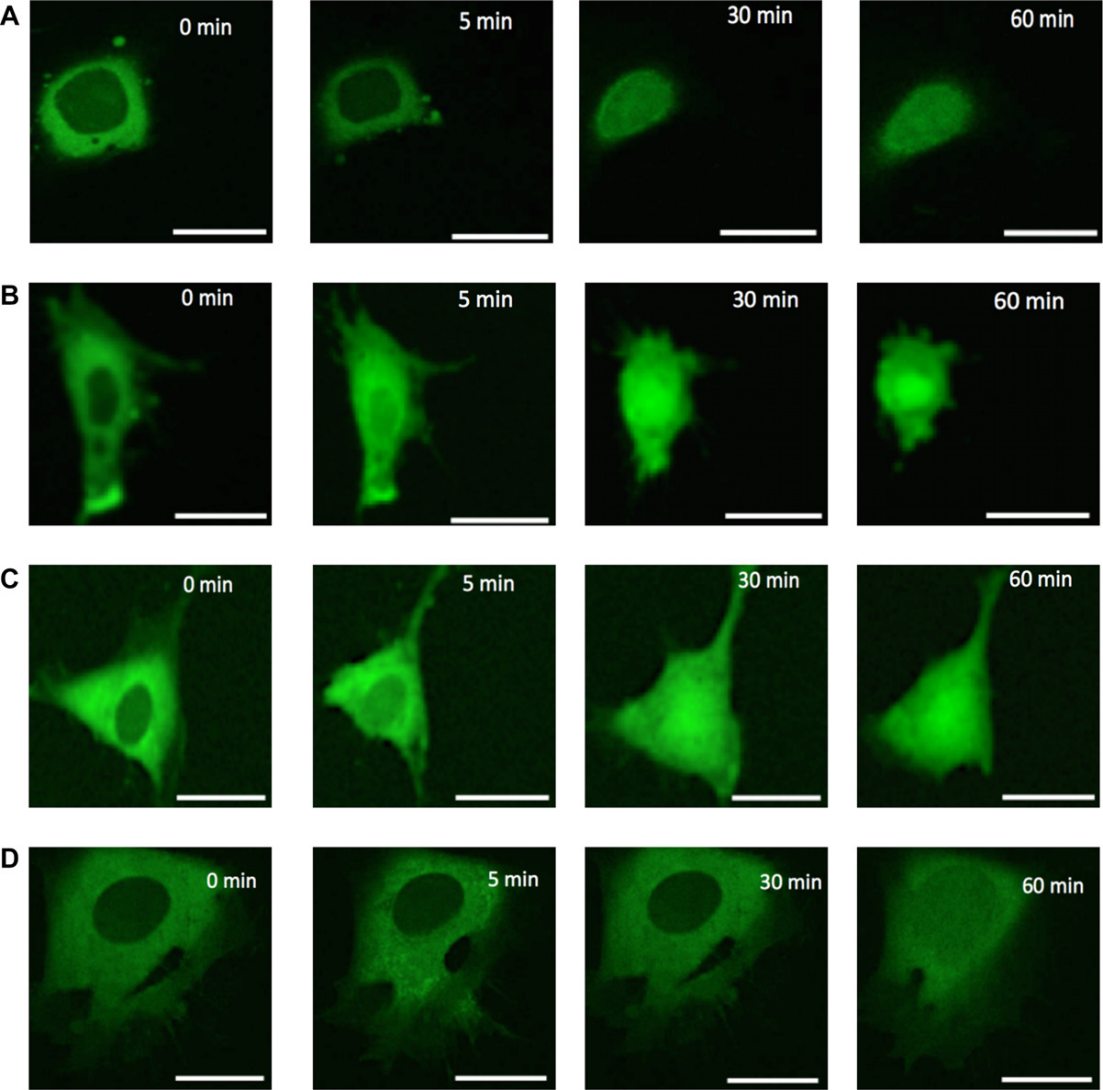
Bulk rhd-tRNA distribution within 0.4 μm-thick confocal slices (located at MEF cells’ mid-height) as a function of time under various nutritional conditions: (**A**) No nutrition, (**B**) 25% nutrition, (**C**) 50% nutrition and (**D**) full (100%) nutrition. Scale bar 25 μm. Time 0 corresponds to <30 s after rhd-tRNA injection.

**Figure 3.**
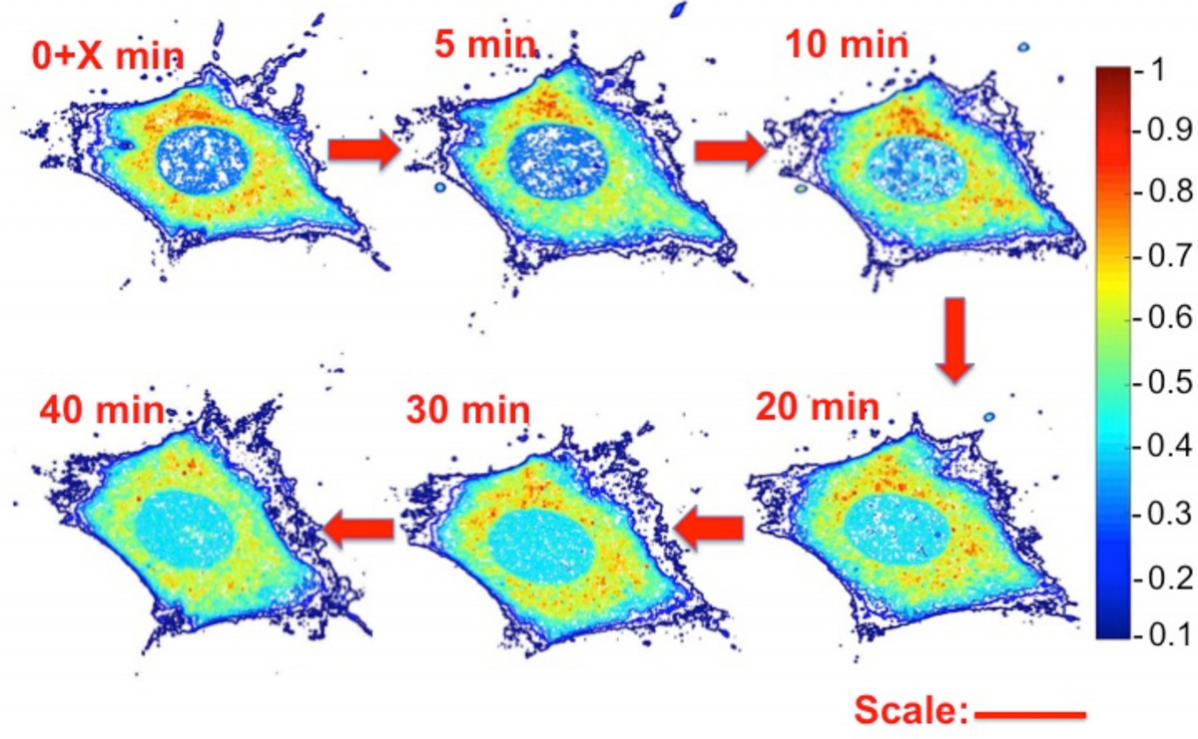
Surface plots of normalized fluorescent emission intensity distribution from a 0.4 μm-thick confocal slice located at cell's mid-height at various times following rhd-tRNA injection. Full nutrition. The emission intensity was normalized with the maximum intensity in the first image. Similar images from confocal slices at the cell's bottom and top are shown in Supplementary Figure SI–S1. Scale bar: 25 μm. Time is measured after *X* < 30 s from tRNA injection into the cytoplasm.

**Figure 4.**
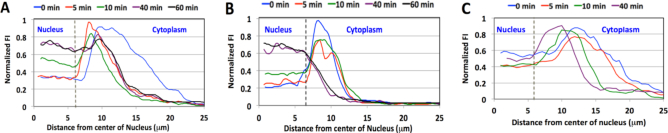
Normalized fluorescence emission intensity along a ray originating at the nucleus center as a function of the distance from the nuclear center at various times after cell injection of the fluorescent-labeled polynucleotides rhd-tRNA (**A, B**) and FAM-80-DNA (**C**) in the presence (100%) (A, C) and absence (0%) (B) of nutrition in the extracellular solution. The vertical line at 7 μm indicates the location of the nuclear membrane. The data is normalized with the initial maximum intensity (at the peak) to compensate for possible variations in illumination and bleaching across experiments.

**Figure 5.**
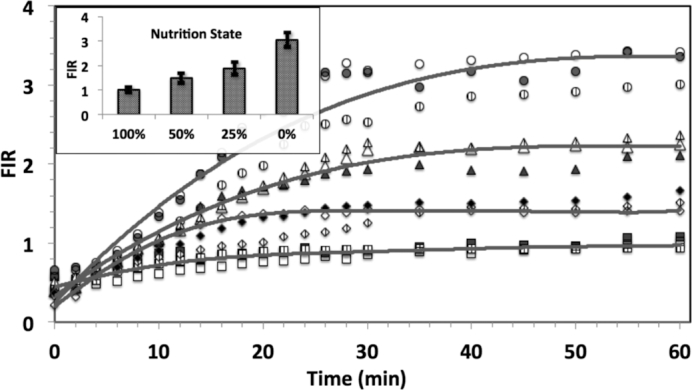
The ratio (*FIR*) of average rhd-tRNA concentration in the nucleus and cytoplasm as a function of time in the presence of 100% (squares), 50% (diamonds), 25% (triangles), and 0% (circles) nutrient. The symbols and solid lines represent, respectively, experimental data for three cells and predictions based on Equation (1). Completely filled, textured, and hollow symbols represent three different cells for each nutrient condition. Cells were maintained in the specified medium for 2 hours prior to rhd-tRNA injection into the cytoplasm. Time *t* = 0 corresponds to <30 s after micro-injection. Inset: A bar graph of the distribution of the steady state FIR_∞_ as a function of nutrition condition. *N* = 20 (full nutrition, no nutrition), 6 (25% nutrition), 5 (50% nutrition).

To detect changes in intracellular tRNA distribution in response to stress, we subjected MEFs to varying degrees of nutritional stress (0–100% normal media in PBS) for 2 h prior to injecting bulk rhodamine-labeled tRNA (rhd-tRNA) into the cytoplasm, and then imaged rhd-tRNA fluorescence emission in these cells for 1 h following injection with a confocal microscope (Figure 1). In all cases (Figures 2–5), rhd-tRNA rapidly (within a minute) accumulated around the nuclear membrane to form a ring of fluorescence, while the nucleus remained relatively dark. As time progressed, nuclear rhd-tRNA concentration increased and the width of the perinuclear ring decreased (Figure 4A), until nuclear rhd-tRNA concentration attained an asymptotic, steady-state value (Figure 5). Interestingly, within our spatial resolution and irrespective of nutritional stress level, fluorescence emission intensity was nearly uniform inside the nucleus, but varied spatially within the cytoplasm, decreasing with increasing radial distance from the nucleus (Figure 4). These observations were carried out within a thin confocal slice and thus were not affected by the variation in the cytoplasm thickness between the perinuclear region and the cell periphery (SI–S2). Similar to the experiments carried out by Plochowietz *et al.* (31), wherein labeled tRNA was introduced into bacterial cells, most of the rhd-tRNA molecules appeared to move freely. A few aggregates of rhd-tRNA, were, however, visible. These may correspond to receptor-bound tRNAs and/or to tRNA liposomes/granules recently reported in neurons (32). The extent of rhd-tRNA nuclear accumulation increased as nutritional stress increased (Figure 5).

We estimated the rate constants for tRNA entry into (*k*_in_, min^−1^) and export from (*k*_out_, min^−1^) the nucleus as functions of the level of nutrition (Table 1) by fitting the fluorescence intensity ratio (FIR = *C*_N_/*C*_C_, Figure 5) of the average nuclear concentration (*C*_N_) and cytoplasmic concentration (*C*_C_) at a distance of 1.5 μm from the nuclear membrane to Equation (1). Equation (1) is derived (SI–S3) from a model which assumes that the cytoplasmic membrane is impermeable to tRNA and that transport processes within the cytoplasm and the nucleus are relatively rapid in comparison to transport through the nuclear envelope, so that the concentration distributions in the nucleus and cytoplasm are at quasi-equilibrium.

(1) \begin{equation*}{\rm FIR}\ \left( t \right) = \frac{{{C_{\rm N}}\left( t \right)}}{{{C_{\rm C}}\left( t \right)}}\ = \frac{{\left( {1 - {e^{ - {\raise0.7ex\hbox{$t$} \!\mathord{\left/ {\vphantom {t \tau }}\right.} \!\lower0.7ex\hbox{$\tau $}}}}} \right)}}{{\left( {\frac{{{k_{{\rm out}}}}}{{{k_{{\rm in}}}}} + \frac{\varphi }{w}\ {e^{ - {\raise0.7ex\hbox{$t$} \!\mathord{\left/ {\vphantom {t \tau }}\right.} \!\lower0.7ex\hbox{$\tau $}}}}} \right)}},\end{equation*}

**Table 1.** Rate constants for different nutritional media (the numbers in parenthesis correspond to standard error)

Nutrition	*k*_in_ (min^−1^)	*k*_out_ (min^−1^)	FIR_∞_ = (*k*_in_/*k*_out_)	τ (min)	*R* ^2 ***^	# of cells analyzed
100% nutrition*	0.30 (0.04)	0.32 (0.04)	1.0 (0.1)	2.40 (0.3)	0.58	20
50% nutrition*	0.16 (0.03)	0.10 (0.02)	1.6 (0.2)	8.8 (2.2)	0.97	4
25% nutrition*	0.18 (0.04)	0.08 (0.03)	2.2 (0.2)	12.6 (2.7)	0.97	6
No nutrition*	0.18 (0.04)	0.06 (0.02)	3.0 (0.5)	14.3(2.0)	0.96	20
100% nutrition + 2 mM puromycin	n.d.	n.d.	0.81 (0.1)	1.06 (0.1)**	0.31	11
no nutrition + 2 mM puromycin	n.d.	n.d.	0.9 (0.06)	1.13 (0.1)**	0.28	5
100% nutrition + 1 min nocodazole	n.d.	n.d.	0.87	9.3 (2.3)**	0.77	11
100% nutrition + 1 h nocodazole	n.d.	n.d.	1.16	10.5 (1.5)**	0.94	7
100% nutrition + 2 h nocodazole	n.d.	n.d.	1.22	10 (1.5)**	0.93	6
100% nutrition + 3 h nocodazole	n.d.	n.d.	1.45	6.8 (1.1)**	0.94	6
100% nutrition + 4 h nocodazole	n.d.	n.d.	1.4	5.5 (0.9)**	0.95	6

* Reaction rate constants determined by fitting data to Equation (1).

** Time constants determined by fitting data to equation SE7.

*** Coefficient of determination [42]

n.d. – not determined. Attempts to fit the data to Equation (1) in the presence of either puromycin or nocodazole failed to converge. The reaction rate constants were determined with equation SI–SE8.

In Equation (1), *w* = $\frac{{{{\bar{C}}_{\rm C}}( t )}}{{{C_{\rm C}}( t )}} \le {\rm{\ }}1$; ${\bar{C}_{\rm C}}( t )$ is the average cytoplasmic tRNA concentration at time *t*; and $\varphi \ = \ \frac{{{V_{\rm N}}}}{{{V_{\rm C}}}}$ is the ratio of the nucleus volume *V*_N_ and the cytoplasm volume *V*_C_.

In all cases, FIR increased with time following injection and eventually approached its asymptotic value FIR_∞_ (inset, Figure 5). Fitting our results to Equation (1) allows estimation of the rate constants of both tRNA import (*k*_in_) and export (*k*_out_). In the absence of pharmaceuticals, both rate constants decrease as the starvation level increases (Table 1), with the decrease in *k*_out_ surpassing the decrease in *k*_in_.

### rhd-tRNA is distributed non-uniformly in the cytoplasm

The results presented in Figures 3 and 4 clearly demonstrate a non-uniform distribution of tRNA in the cytoplasm within the mid-height confocal slice at all levels of nutrition. To better characterize rhd-tRNA spatial distribution in the cell, we quantified rhd-tRNA emission intensities along radial lines originating at the center of the nucleus and extending to the cell periphery. Figure 4A and B depict, respectively, normalized rhd-tRNA emission intensity as functions of the distance from nucleus’ center (*r*) at various times following rhd-tRNA injection into the cytoplasm at 100% (A) and 0% (B) nutrition. In all cases, fluorescence emission intensity inside the nucleus increased with time. The increase was greater at 0% nutrition (FIR_∞_ ∼ 3) than at 100% nutrition (FIR_∞_ ∼ 1) (inset, Figure 5).

At 100% nutrition, the ring of rhd-tRNA surrounding the nucleus (Figure 4A) exhibited a prominent peak of fluorescence intensity. From this peak, intensity decreased as the radial distance from the nuclear membrane increased, in a pattern that was maintained for the entire 60 min following rhd-tRNA injection. This pattern was also seen at ≤10 min following rhd-tRNA injection at 0% nutrition, but at ≥40 min the ring of peak rhd-tRNA emission intensity disappeared, and emission intensity in the cytoplasm decreased monotonically with radial distance. Similar behaviors were observed in the bottom and top confocal slices (Supplementary SI–Figure S1).

To determine whether the non-uniformity in cytoplasmic tRNA distribution is specific to tRNA or common to fluorescently-labeled polynucleotides, we injected a fluorescently labeled 80 nt ssDNA (FAM-80-DNA, SI S7), similar in length to tRNA, into cells maintained at 100% nutrition and assessed ssDNA concentration distribution in the cell as a function of time (Figure 4C). We hypothesize that an ssDNA is less likely to have specific interactions with cytoplasmic components than tRNA. The ssDNA distribution in the cytoplasm at full nutrition appeared qualitatively similar to that of tRNA (Figure 4A). Thus, tRNA distribution in the cytoplasm, unlike its accumulation in the nucleus, does not appear to be due to a tRNA-specific mechanism.

### Cells react rapidly to nutrition deprivation

**Figure 6.**
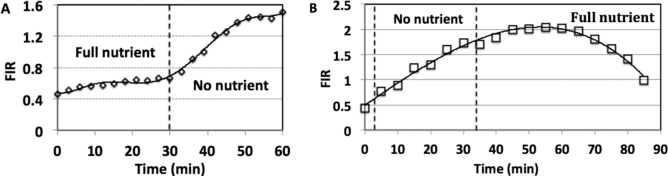
Cytoplasmic tRNA shuttling between nucleus and cytoplasm when the cell media is changed during observation. (**A**) Cells are deprived of nutrition by replacing fresh media with PBS after 30 min of observation, resulting in retrograde nuclear accumulation (*N* = 27). (**B**) The cell is replenished with fresh media after 30 min in PBS (0% nutrition), resulting in tRNA export from the nucleus to the cytoplasm (*N* = 6).

In our experiments described above, we maintained cells in a specific extracellular solution for 2 h, allowing them to attain near-equilibrium with the extracellular environment, before injecting rhd-tRNA into the cytoplasm. To examine how fast the cell responds to variations in its environment, we injected rhd-tRNA into cells in a 100% nutrition medium and after 30 min changed the medium to PBS solution (0% nutrition). At 100% nutrition, tRNA accumulated in the nucleus, attaining FIR ∼0.7 after 30 min (Figure 6A). This value is smaller than FIR_∞_ since the time of initial incubation was less than that required to establish equilibrium. When nutrients were removed from the extracellular solution, FIR increased to >1.0 within <10 min of nutrition-deprivation, demonstrating the rapidity with which cells react to nutrition deprivation.

### Retrograde tRNA transport is reversible

Results presented above demonstrate that depriving cells of nutrients induces tRNA retrograde translocation into the nucleus. To examine the reversibility of this process, we injected rhd-tRNA into the cytoplasm of starved cells, leading, as expected, to an initial increase in FIR (Figure 6B, left). However, when, after 33 min, the extracellular medium was furnished with 100% nutrient, FIR decreased after a short delay of ∼20 min (Figure 6B, right), approaching its initial value as the cells recovered from starvation to full nutrition. These results are qualitatively consistent with our model predictions (SI–S5), making the simplifying assumptions that *k*_in_ and *k*_out_ increase linearly from their starvation values over time interval of 25 min, after which they reach their full nutrition values (Supplementary Figure S5).

### Puromycin suppresses starvation-induced retrograde tRNA accumulation in the nucleus

**Figure 7.**
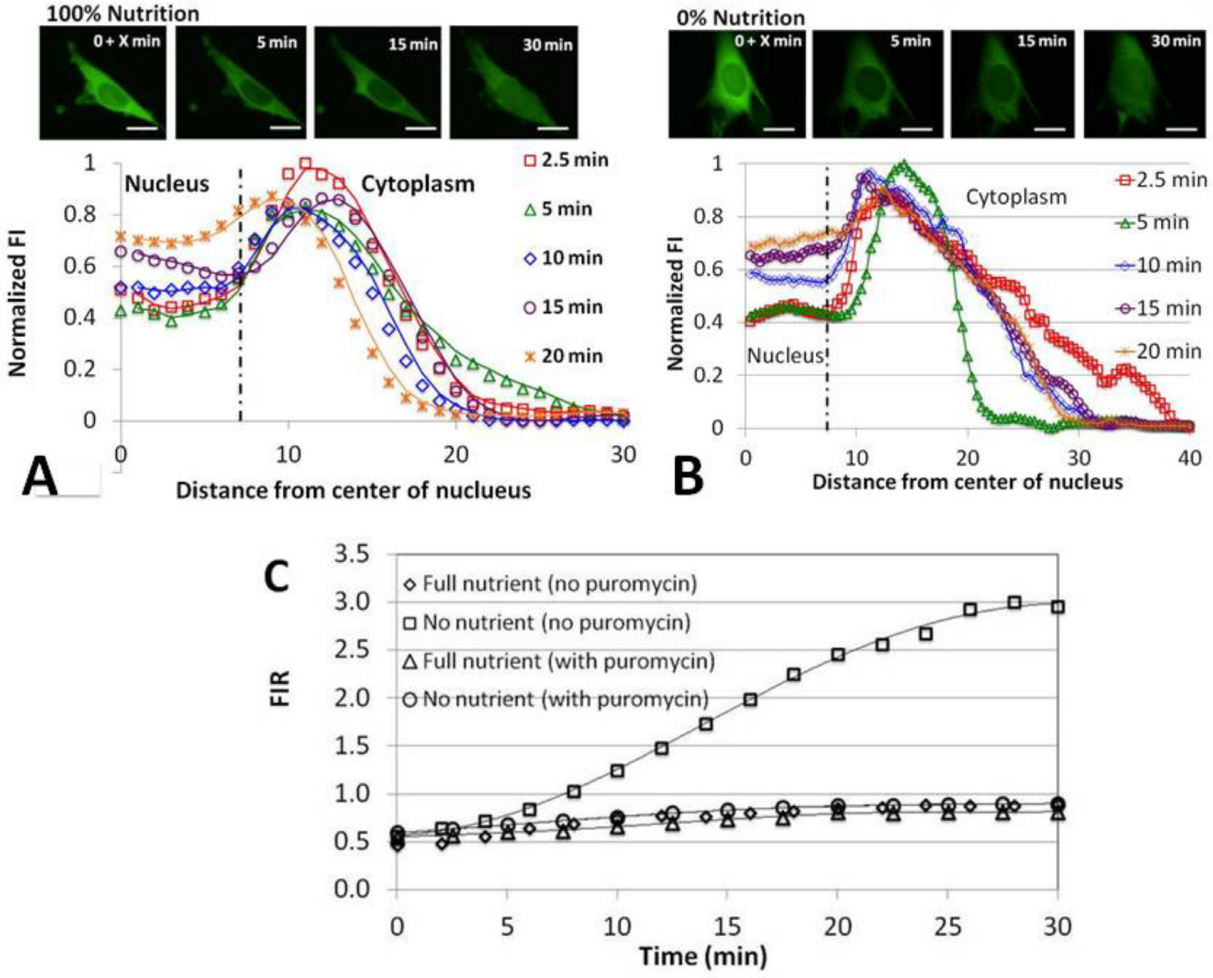
Puromycin suppresses nuclear tRNA aggregation (see SI-S6 for enlarged images). Normalized fluoresence intensity as a function of distance from the nucleus center in the presece of puromycin and 100% (**A**) or 0% (**B**) nutrition. (**C**) FIR as a function of time in the presence (*N* = 11) and absence (*N* = 5) of puromycin and in the presence and absence of nutrition. *X* < 30 s.

To test whether tRNA translocation into the nucleus is dependent on active protein synthesis, we co-injected cells with 2 mM of the translation inhibitor puromycin concurrently with rhd-tRNA and monitored rhd-tRNA distribution for 30 min after injection, in the presence (100%, Figure 7A) and absence (0%, Figure 7B) of nutrition (see also SI–S6). Both cases exhibit the characteristic non-uniform spatial tRNA distribution in the cytoplasm. In the presence of puromycin at both 100% and 0% nutrition, the nuclear tRNA concentration increases with time, but never exceeds the maximum tRNA concentration in the cytoplasm. Figure 7C compares FIR in the presence and absence of puromycin and in the presence and absence of nutrition. In the presence of puromycin, FIR_∞_ was nearly unaffected by nutrition level (Table 1) while in the absence of puromycin, FIR_∞_ increased from ∼1 to ∼3 as nutrition level depleted from 100% to 0%. Our results differ from those presented in a prior report (29) showing that FIR increases about 2 fold when Chinese hamster ovary cells (CHO) transfected with Cy3-labeled tRNA are treated with puromycin under full nutrition conditions. We have not observed such a pronounced effect under full nutrition, perhaps due to differences in cell types and/or internal rhd-tRNA concentrations in the two experiments. We were unable to estimate reaction rate constants with Equation (1). In Table 1, we report only time constants based on simple exponential fits (SI-SE8). Interestingly, by this criterion puromycin appears to accelerate tRNA translocation at both 100% and 0% nutrition, suggesting that puromycin treatment may lead to a selective decrease in the levels of one or more proteins that down-regulate tRNA nuclear transport.

### Cell exposure to nocodazole enhances tRNA transport rate

**Figure 8.**
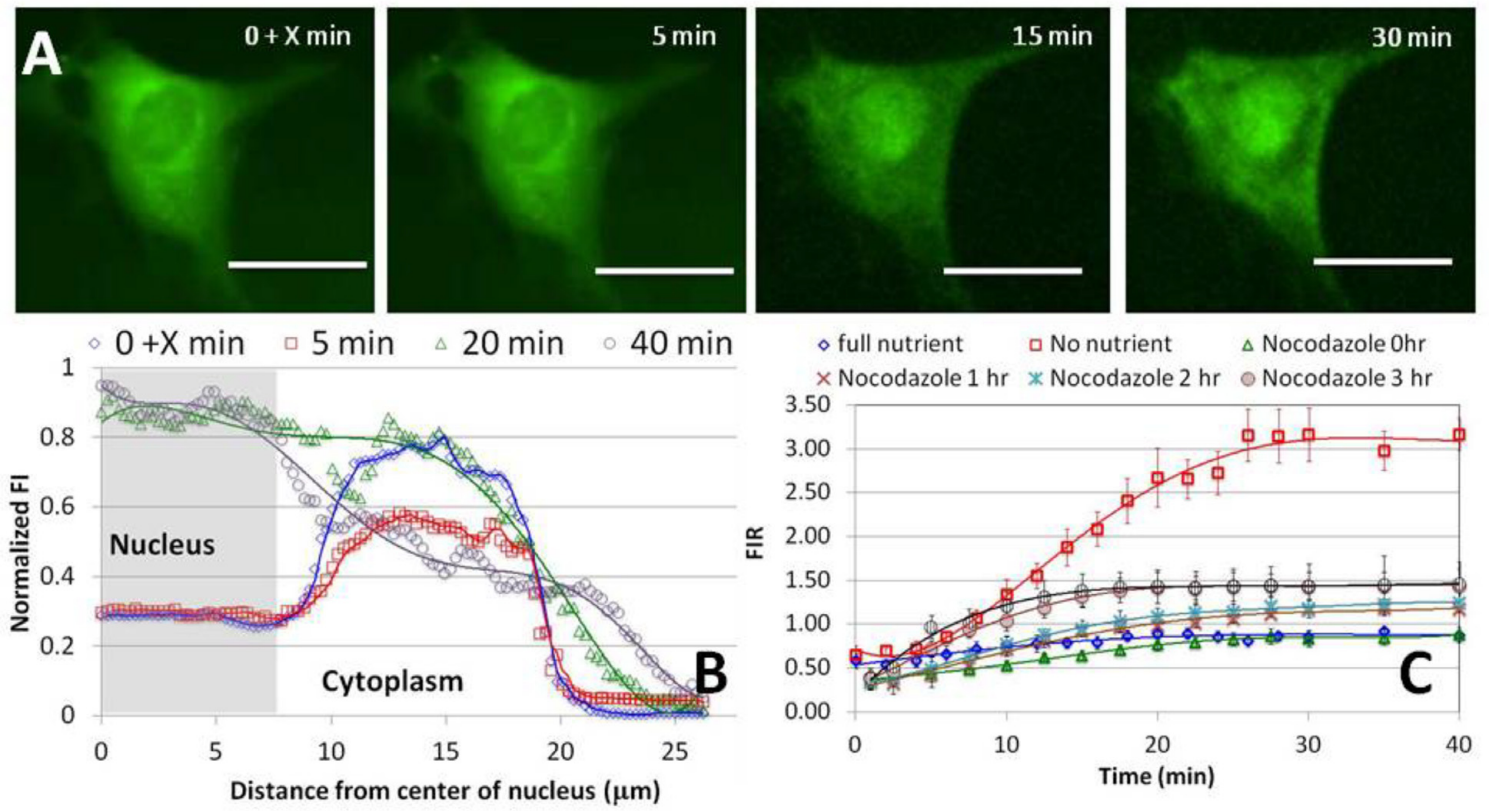
(**A**) rhd-tRNA emission intensity from a 0.4 μm confocal slice of a cell pre-exposed for 3 h to 100 nM extracellular microtubule de-polymerizing agent nocodazole. Scale bar 25 μm. X < 30 *s* corresponds to time after injection. See SI-S7 for additional data. (**B**) Fluorescent intensity along the cell's diameter at various times after rhd-tRNA injection into the cell. (**C**) Fluorescent intensity ratio (FIR) as a function of time under 100% and 0% nutrition in the absence of nocodazole, and under 100% nutrition and 0.02 (*N* = 11), 1 (*N* = 7), 2 (*N* = 8), 3 (*N* = 6) and 4 (*N* = 6) hours pre-exposure to nocodazole.

To examine whether tRNA transport into the nucleus and tRNA spatial distribution in the cytoplasm depend on active transport along microtubules, previously implicated in transport of tRNA-containing granules in neurons (32), we injected bulk rhd-tRNA into cells that had been pre-treated with the microtubule depolymerizing drug nocodazole (33) (100 nM, added to the extracellular media at 100% nutrition) for up to 4 h (SI–S7). We determined the radial distribution of tRNA emission intensity from a 0.4 μm thick confocal slice at cell's mid-height at various times, up to 40 min after rhd-tRNA injection (Figure 8A). The tRNA concentration distribution in the cytoplasm remains non-uniform after microtubule depolymerization, indicating that active transport by protein motors is not the principle cause of non-uniform tRNA distribution within the cytoplasm.

As compared with cells at 100% nutrition not exposed to nocodazole, FIR_∞_ increased monotonically from a value of ∼1 as the pre-exposure time to nocodazole increased, reaching an apparent plateau value of ∼1.4 in cells with over three hours pre-exposure to nocodazole (Figure 8B and Table 1). The time constant of retrograde transport shows complex behavior, rapidly increasing by ∼4-fold (9.3 min versus 2.4 min) in cells exposed to nocodazole during tRNA translocation, and decreasing thereafter to 5.5 min after 4 h of pre-exposure to nocodazole.

## DISCUSSION

Our study monitors, for the first time, the kinetics of tRNA nuclear import and export in an experimental system in which tRNA subcellular dynamics can be isolated from tRNA transcription, and which is amenable to measurements in live cells. We monitor in real time the kinetics of tRNA trafficking between the cytoplasm and the nucleus following controlled injection of fluorescent rhd-tRNA into the cytoplasm of single live cells. Our results improve our understanding of the behavior of intracellular tRNA. Our approach, combining microinjection with fluorescent microscopy, provides a new way to study the diverse roles of these important biological molecules, and is applicable to address other important questions in cell biology.

### In the absence of nutrition, tRNA accumulates rapidly and reversibly in the nucleus

Our results provide clear evidence that following injection of tRNA into the cytoplasm, accumulation of tRNA in the nucleus proceeds via a rapid (within minutes) and reversible process. Application of a simple kinetic model allows us to estimate the import and export reaction rate constants (Supplemental Information S3). At 100% nutrient, the nuclear rhd-tRNA concentration eventually attains a level similar in magnitude to that just outside the nuclear membrane (FIR_∞_ ∼ 1) and the rate constants for tRNA export out of and import into the nucleus are similar (Table 1). Both rates decrease as the nutrient level is lowered, but the effect is much greater on the export rate, with the resulting imbalance leading to an FIR_∞_ of three under complete starvation (Figure 5, inset). This result is consistent with data showing net retrograde transport of tRNA under amino acid, glucose, and inorganic phosphate starvation in *S. cerevisiae* (2,7,28). Since all rhd-tRNA is initially injected into the cytoplasm, an FIR greater than one forces the conclusion that, at least at low nutrition, accumulation of tRNA in the nucleus cannot be accomplished passively via nuclear pores and must require active pumping. When the high nutrition extracellular medium is refurnished, tRNA migrates reversibly and rapidly from the nucleus to the cytoplasm and FIR decreases to about one (Figure 6B), consistent with our model predictions (SI–S5).

### Pharmaceuticals modify tRNA import and export

Both tRNA import and export are known to proceed via specific protein carriers (3), making it somewhat unexpected that the translation inhibitor puromycin suppresses nuclear tRNA accumulation under starvation conditions (Figure 7 and Table 1) and maintains FIR_∞_ ∼ 1 in the absence (0%) of nutrition. The detailed mechanisms underlying the rate results reported in Table 1 and the role of puromycin remain to be elucidated. Equally unclear are the detailed mechanisms giving rise to the effects of the depolymerizing drug nocodazole, but some speculations are warranted. The rapid rise in the time constant for transfer seen in cells exposed to nocodazole (Table 1) suggests that protein motors may participate in tRNA transport in the cytoplasm, as demonstrated recently in neural cells (32). On the other hand, the subsequent decrease in time constant that coincides with extensive depolymerization, could be attributable to increased cytoplasmic rates of tRNA diffusion and migration, and implies the existence of tRNA transport mechanisms other than protein motors. Protein motors are not the primary mechanism that maintains the non-uniformity of tRNA cytoplasmic concentration distribution, since this distribution is little affected by prolonged nocodazole treatment.

### Non-uniform spatial distribution of tRNA in the cytoplasm

We consistently observe a non-uniform concentration distribution of rhd-tRNA between the perinuclear region and the cell periphery at cell's midheight. At short times, the cytoplasmic tRNA concentration peaks a short distance from the nuclear membrane and declines towards the plasma membrane (Figure 4). The height of the peak relative to the nuclear tRNA concentration decreases as time increases. If diffusion alone were responsible for cytoplasmic tRNA transport, we would expect the rhd-tRNA concentration following injection to decline as we approach the nuclear envelope; we see the opposite. Furthermore, at equilibrium, one would expect a uniform tRNA concentration throughout the cytoplasm. Clearly, this is not the case. Rather, the cytoplasmic tRNA concentration decreases as the distance from the nucleus increases (Figure 4).

We exclude imaging artifacts (SI–S4), leakage of tRNA through the plasma membrane (SI S5), and protein motors as primary mechanisms for non-uniform tRNA distribution. So what causes non-uniform cytoplasmic tRNA distribution? Below, we propose two plausible mechanisms that might be responsible.

### Proposed mechanism 1 for nonuniform tRNA distribution: Specific tRNA interactions

tRNA interactions in the cytoplasm could cause non-uniform distribution, with a tendency to concentrate tRNA near the nucleus. Consistent with this mechanism is the spatial distribution of hot spots, representing tRNA aggregates (Figure 3 and Supplementary Figure SF2). Such hot spots could be due to tRNA binding to cell components, such as polysomes and multi-aminoacyl-tRNA synthetase complexes (MSCs). Indeed, MSCs are recruited by stress-granules, a depot for mRNA and translation components (32). Alternatively, or in addition, the hot spots could represent tRNA granules, distinct from stress granules, that have recently been reported when tRNAs are transfected into neuroblastoma cells by either electroporation or lipofection (32). However, the similarities in the non-uniform cytoplasmic distributions of FAM-80-DNA and rhd-tRNA (Figure 4) argue against a tRNA-specific mechanism as being solely responsible for the observed non-uniformity.

### Proposed mechanism 2 for non-uniform tRNA distribution: Electrophoresis

A mechanism that would be common to all polynucleotides is migration in an electric field (electrophoresis). An electric field directed from the nucleus vicinity outwards within the confocal slice at cell's midheight would apply an electrostatic force to the negatively-charged polynucleotides, causing them to migrate towards the nucleus. Such a field might arise from ionic pumps in the plasma and/or nuclear membranes that induce ionic currents in the cytoplasm. Although cell injury resulting from microinjection might induce an electric field in the cytoplasm (34), such an electric field would be short–lived, lasting a few minutes, inconsistent with the lengthy steady-state we observed.

The two mechanisms considered above are not mutually exclusive. The rough ER is near the nucleus. High local tRNA concentration may be caused by interactions with the ribosomes in the rough ER, especially at full nutrition, explaining the concentration peaks in Figure 4. The presence of the peak in tRNA concentration outside the nuclear membrane can also be attributed to variations in the cytoplasm's electric properties. Interestingly, the electric field may assist in protein synthesis by counteracting diffusion and concentrating tRNA next to the ribosome.

Our results strongly support the notion that, in starving cells, tRNA trafficking across the nuclear envelope is actively controlled, is rapid (within minutes), and reversible. The dependence of import and export rate constants on nutrition level suggests that ATP levels might mediate this control. The process is modulated by pharmaceuticals - retrograde tRNA nuclear accumulation is suppressed in cells treated with puromycin and enhanced in cells treated with nocodazole.

Lastly, we have demonstrated that our results can plausibly be explained by the hypothesis that even a non-excitable cell can function as a battery, converting chemical energy (ATP) to electric energy via ionic pumps that generate electric fields and polarize the cell to support a biological process. We believe that such an admittedly speculative hypothesis is worth pursuing.

## Supplementary Material

Supplementary Data
