# Supplementary material for: Dynamics of intracellular stress-induced tRNA trafficking
Source: Nucleic Acids Res. 2018 Nov 28;47(4):2002–10. doi: 10.1093/nar/gky1208 (PMC6393242; doi:10.1093/nar/gky1208)
Supplement: Supplementary Data [file gky1208_supplemental_files.pdf]

Dynamics of Intracellular Stress-Induced tRNA Trafficking

Supporting Information

Rabin Dhakal[1], Chunyi Tong[1,*], Sean Anderson[1], Anna S. Kashina[2], Barry Cooperman[3], and Haim H, Bau**[,1]

1. Department of Mechanical Engineering and Applied Mechanics, University of Pennsylvania, Philadelphia, PA 19104

2. Department of Biomedical Sciences, School of Veterinary Medicine, University of Pennsylvania, Philadelphia, PA 19104

3. Department of Chemistry, University of Pennsylvania, Philadelphia, PA 19014

*Current address: College of Biology, Hunan University, Changsha, 410082, P.R. China

** Corresponding author: bau@seas.upenn.edu, 215-898-8363

S1. Spatial distribution of tRNA emission from confocal slices next to the cell's bottom, mid-height, and top

The fluorescence emission intensity distribution is nearly uniform inside the nucleus and varies spatially in the cytoplasm with a rapid radial decrease from a maximum value outside the nuclear envelope towards the cell membrane, irrespective of nutritional stress level. **Fig. S1** depicts surface plots of the normalized florescence intensity as a function of time at cell mid-height, bottom, and top. The positions of the bottom, mid-height, and top confocal slices are specified in **Fig. 1**. The boundary between the nucleus and cytoplasm is clearly visible. We were not able to image tRNA distribution in the space above the nucleus due to the small gap between the top of the nucleus and the top of the cell.

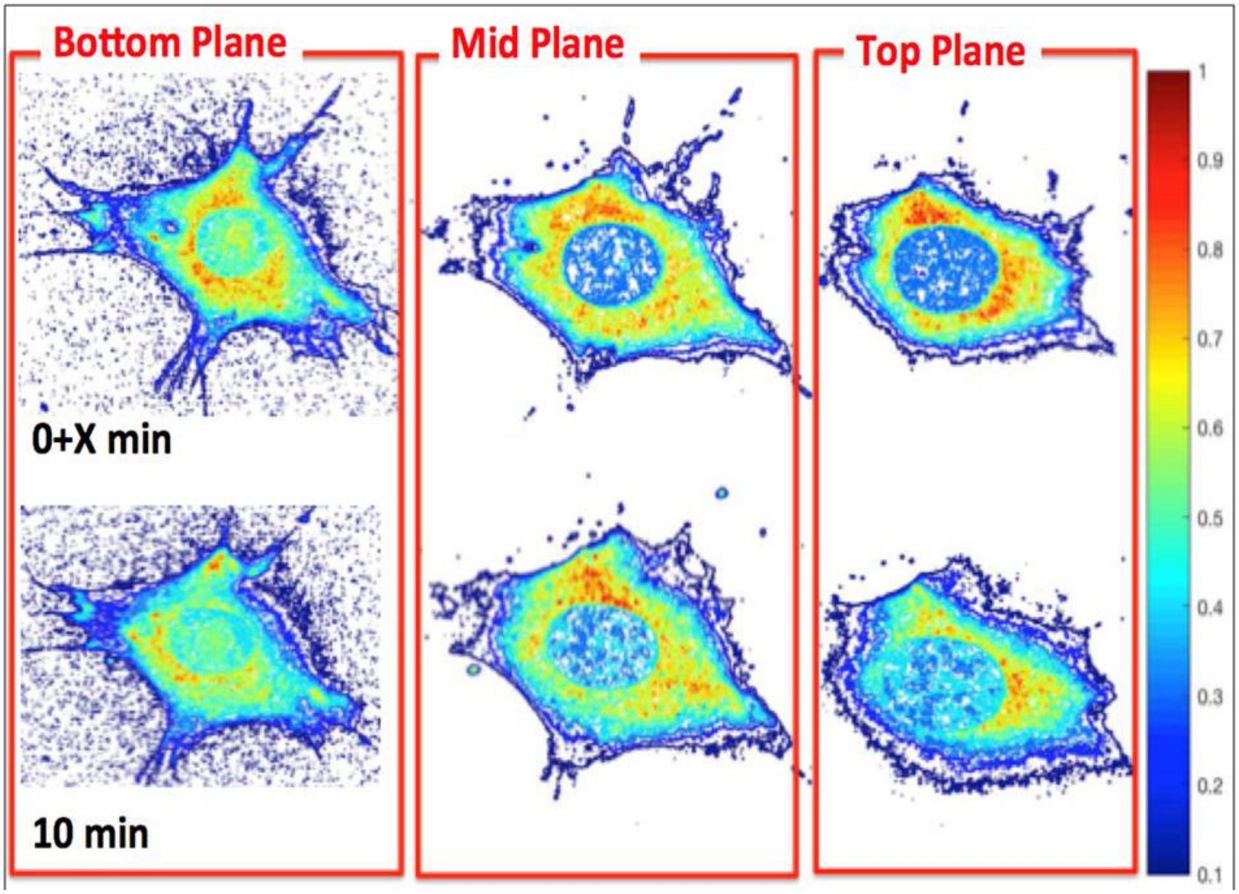

Fig. S1: Surface plots of the normalized fluorescence intensity distributions from confocal slices at cell's bottom, midplane, and top (as defined in Fig. 1) 15s and 10 min after injection. The color bar represents the normalized intensity value. Full nutrition.

A ring of high rhd-tRNA emission intensity surrounds the nucleus a short distance from the nuclear membrane. The intensity decreases towards the cell membrane. This non-uniform distribution is maintained during the entire observation time. The peak intensity ring is not

continuous and islands of high concentration are visible, possibly due tRNA accumulation in organelles or vesicles. The intensity plots of the bottom confocal slice exhibit noisy background, likely due to proximity to the substrate.

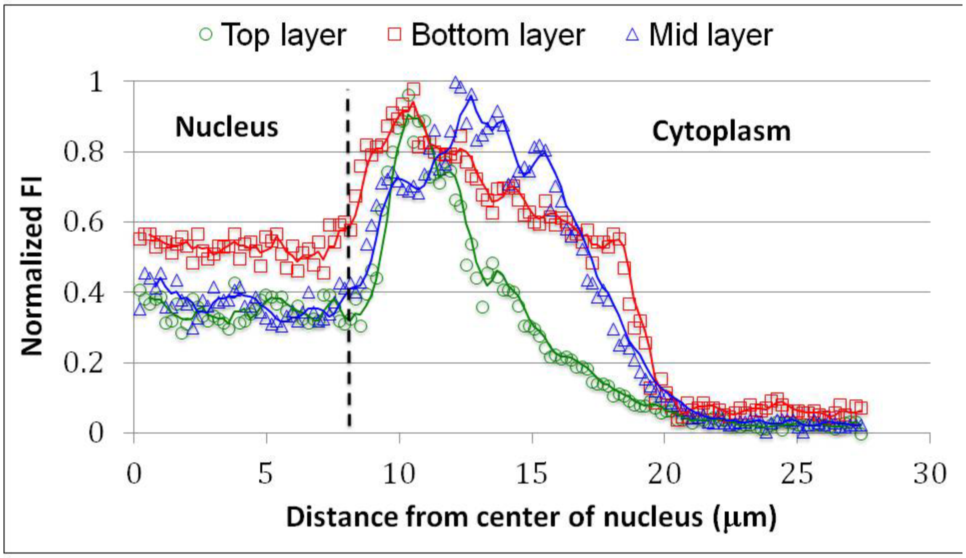

Fig. S2: Radial distribution of the normalized FI as a function of distance from center of nucleus for three different confocal layers immediately after injection. The thickness of each confocal slice is 500 nm. Full nutrition.

Fig. S2 depicts the radial distribution of the normalized fluorescence emission intensity (FI) from the bottom, midheight, and top confocal slices. tRNA is nearly uniformly distributed inside the nucleus. The intensity peaks a few micrometers from the nuclear membrane. FI gradually decreases towards the cell membrane in all three confocal slices, exhibiting non-uniform distribution.

S2. Effect of confocal slice thickness on fluorescence emission intensity

Since the height of the surface-mounted cell is maximal at the nucleus position and declines as the distance from the nucleus (towards the plasma membrane) increases (**Fig. 1**), there is a concern that cell geometry could affect emission intensity. To examine for potential effects of cell geometry, we examined fluorescence emission from confocal slices of different thicknesses – all located at cell midheight, **Fig. S3** depicts emission intensities of mid-height confocal slices of thicknesses of 400, 600, 800, and 1000 nm as functions of distance from the nucleus' center. The four intensity curves nearly overlap. We conclude that cell's geometry does not significantly affect our data.

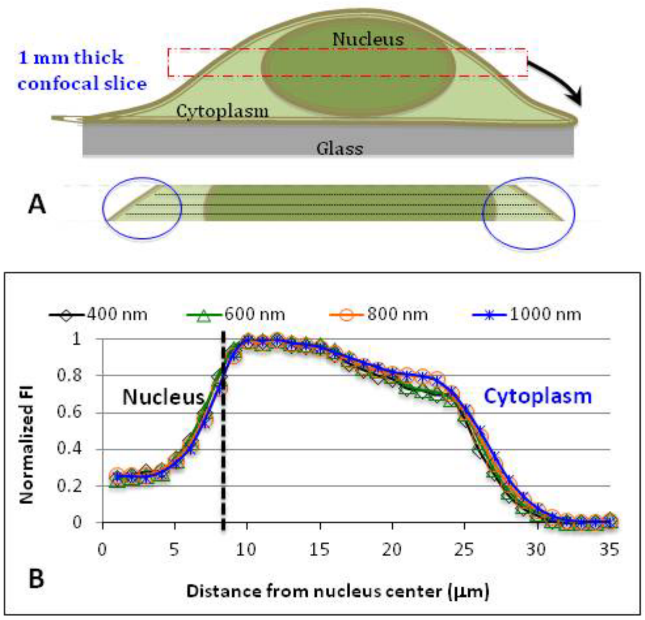

Fig. S3: Normalized emission intensities as functions of distance from the nucleus center when the thickness of the confocal slice is 400, 600, 800, and 1000 nm short time after injection. (**A**) A schematic showing 1 μm thick mid plane confocal slice (top) and subslices (bottom). (**B**) Normalized fluorescent emission intensity FI as a function of distance from center of nucleus for the four different confocal slice thicknesses.

S3. A model for estimating rate constants of tRNA translocation

To estimate the reaction rate constants of tRNA translocation through the nuclear envelope, we formulate a simple lumped-parameter kinetic model for nuclear / cytoplasmic trafficking. Our model assumes that the transport through the nuclear envelope is the rate-limiting step; transport processes in the cytoplasm and the nucleus are relatively rapid in comparison, so that the concentration distributions in the nucleus and cytoplasm are at quasi-equilibrium. We assume that the cytoplasmic membrane is impermeable to tRNA. As in the main text, we define the fluorescent intensity ratio $FIR(t)= C_N(t)/C_C(t)$. Since tRNA distribution in the nucleus is nearly uniform (at the resolution of our microscope), it is reasonable to use the average nuclear concentration for $C_N(t)$ (mol/m^3). Since transport kinetics through the nuclear envelope is controlled by the tRNA concentration just outside the nuclear envelope, we use for $C_C(t)$ the concentration in the cytoplasm outside the nuclear envelope (**Fig. 1**). We have, also calculated the reaction rate constants when $C_C(t)$ is replaced with the average tRNA concentration in the cytoplasm $\bar{C}_C(t)$. $C_C(t)$ differs from the average cytoplasmic concentration $\bar{C}_C(t)$ even under steady state conditions– for reasons discussed in the main text.

We describe the rhd-tRNA mass flux J_i (mol/m[2]-s) through the nuclear membrane with the first order kinetic model:

$$J_{out} = \lambda_{out} C_N \text{ and } J_{in} = \lambda_{in} C_C. \tag{SE1}$$

In the above, J_{in} and J_{out} are, respectively, the tRNA molar fluxes (mol/m^2-s) in and out of the nucleus. λ_{in} and λ_{out} are, respectively, the rate constants (m/s) for transport into (import) and from (export) the nucleus.

Mass transport balance in the nucleus:

$$V_N \frac{dC_N}{dt} = A\lambda_{in} C_C - A\lambda_{out} C_N \tag{SE2}$$

and in the cytoplasm:

$$V_C \frac{dC_C}{dt} = A\lambda_{out} C_N - A\lambda_{in} C_C. \tag{SE3}$$

In the above, A is the effective surface area of the nuclear membrane through which tRNA exchange takes place; V_C and V_N are, respectively, the volumes of the nucleus and cytoplasm. In our experiment, we did not observe any dye leaching from the cell. Hence, we assume that the cell membrane is impermeable to tRNA. Thus, at any instant in time,

$$C_N V_N + wC_C V_C = C_o(V_N + V_C), \tag{SE4}$$

where $C_o(V_N + V_C)$ is the total amount of tRNA injected into the cell. *w≤1* is a weighing factor relating the average cytoplasmic tRNA concentration to C_c. $\bar{C}_C(t) = wC_C(t)$. When *w=1*, C_c assumes the role of average, cytoplasmic concentration. We confirmed that *w* is a weak function of time (quasi-static approximation). C_o is the cell-averaged tRNA concentration. The initial conditions are:

$$C_N(0) = C_{N0} \text{ and } C_C(0) = C_o \frac{(1+\phi)}{w}, \tag{SE5}$$

where $\phi = \frac{V_N}{V_C}$ is the ratio between the nuclear volume and the cytoplasm volume. Further, we neglect variations in cell geometry on the time scale of our experiment and treat V_N, V_C, A/V_N, k_{in}, k_{out}, and *w* as time-independent (quasi-static approximation).

$$FIR(t) = \frac{C_N(t)}{C_C(t)} = \frac{FIR_0\left(\frac{k_{out}}{k_{in}} e^{-t/\tau} + \frac{\varphi}{w}\right) + \left(1 - e^{-t/\tau}\right)}{\left(\frac{k_{out}}{k_{in}} + \frac{\varphi}{w} e^{-t/\tau}\right) + FIR_0\left(\frac{k_{out}}{k_{in}}\right)\left(\frac{\varphi}{w}\right)\left(1 - e^{-t/\tau}\right)} \tag{SE6}$$

Conveniently, *FIR* is independent of the mass of injected tRNA. In the above, $\tau = \left(k_{out} + k_{in}\frac{\varphi}{w}\right)^{-1}$ approximates the relaxation time constant, $k_i = \frac{A}{V_N}\lambda_i$ (*min*$^{-1}$), and $FIR_0 = \frac{C_{N0}}{C_C(0)}$. Strictly speaking, τ, φ , and *w* and possibly k_{in} and k_{out} are time-dependent.

At long times ($t \rightarrow \infty$), consistent with our experimental data, *FIR* attains the asymptotic value $FIR_\infty = \frac{k_{in}}{k_{out}}$.

In our first set of experiments, immediately after injection, all the rhd-tRNA is in the cytoplasm. $C_N(0)= FIR(0)=FIR_0=0$, and we obtain equation (1) of the main text

$$FIR(t) = \frac{C_N(t)}{C_C(t)} = \frac{\left(1-e^{-t/\tau}\right)}{\left(\frac{k_{out}}{k_{in}}+\frac{\varphi}{w}e^{-t/\tau}\right)}. \qquad \text{(SE7)}$$

To estimate the kinetic rate constants: k_{in} and k_{out} for the various nutrient conditions, we minimize the squared difference $\Psi\left(k_{in},k_{out}\right)=\int_0^{t_1}\left(FIR_{th}\left(k_{in},k_{out},t\right)-FIR_{\exp}\left(t\right)\right)^2 dt$ between our experimental data (**Fig. 4**) and our model predictions over the two-dimensional parameter space k_{in} and k_{out}. Although it is impossible to determine *w* precisely, our calculations suggest that predicted reaction rate constants are insensitive to the value of w.

Fig. 4 depicts landscape plots of $\Psi(k_{in}, k_{out})$ as a function of k_{in} and k_{out} for various nutrient conditions. The data suggests that in all these cases of nutrition deprivation, $\Psi(k_{in}, k_{out})$ is convex with a single minimum.

Table 1 of the main text lists the estimated k_{in}, k_{out}, $FIR_\infty=k_{in}/k_{out}$, relaxation time constant τ, and R^2 for various nutrient conditions and the model based on $C_c(t)$. The computed reaction rate constants do not vary considerably when C_c is replaced with the average cytoplasmic concentration. The model is robust and the estimated reaction rate constants depend only weakly on φ/w.

Typically, $\varphi \sim 0.1$ [SR1]. When $\frac{\varphi}{w} \ll 1$, equation (SE6) simplifies to

$$FIR(t) = \frac{C_N(t)}{C_C(t)} = FIR_\infty\left(1-e^{-t/\tau}\right) \qquad \text{(SE8)}$$

and $\tau \sim (k_{out})^{-1}$.

To check the validity of our assumption that transport in the cytoplasm is relatively rapid compared to the rate of translocation through the nuclear envelope, we compare the time constants associated with mass transport by diffusion in the cytoplasm $\tau_{Diffusion} = \frac{\left(r_C - r_N\right)^2}{D_{tRNA}} \sim 0.7\min$ with the time constants associated with translocation (smallest time constant in the absence of either puromycin or nocodazole, 2.4 min, full nutrition, Table 1). The mass transfer in the cytoplasm is, indeed, faster than the translocation time, justifying our quasi-static approximation.

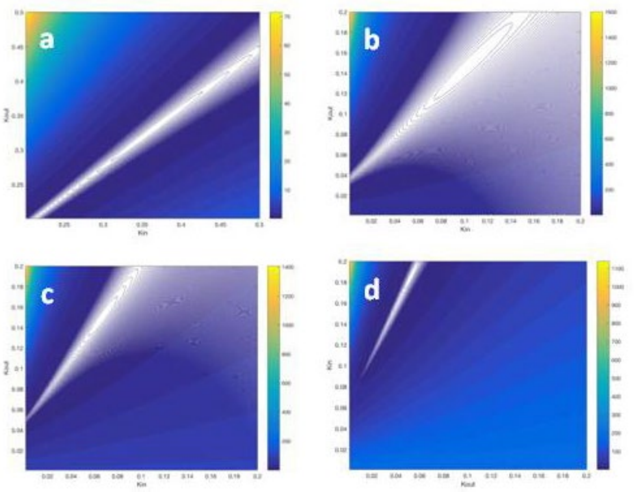

Fig. S4: Landscape plots of the discrepancy between experimental data and theoretical predictions as functions of rate constants. (a) 100% Nutrition, (b) 50% nutrition, (c) 25% nutrition and (d) 0% nutrition conditions. The color bar represents the mean square error value. The curves represent contours of fixed discrepancy.

S4. tRNA leakage through the plasma membrane

Another potential artifact that could cause non-uniform tRNA concentration distribution in the cytoplasm is tRNA leakage through the plasma membrane. We did not, however, observe any presence of dye in the extracellular solution. Additionally, the establishment of an eventual steady-state tRNA distribution (**Fig. 5**) excludes the possibility of any significant tRNA leakage through the plasma membrane.

S5. Reversibility of tRNA retrograde transport

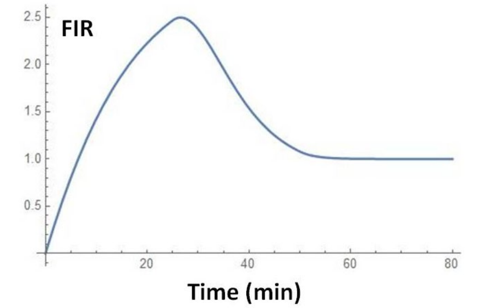

Fig. S5: Predictions of FIR as a function of time in a cell starved when t<25min and exposed to nutrient when t>25 min. The reaction rate constants are assumed to recover their full nutrition values within 25 min.

In our experiments (Fig. 6B), we observe that tRNA injected into the cytoplasm of starved cells aggregated in the nucleus and *FIR* increased. Once the extracellular media has been refurnished with nutrient, *FIR* decreased again. We surmise that the nutrients restore gradually the reaction rate constants (**Table 1**) to their values at full nutrition. To demonstrate that our model predicts qualitatively the experimentally-observed phenomenon, we integrated equations (SE2) and (SE3) with the initial condition $C_N(0)=0$. We kept k_{in} and k_{out} at

their starvation values when 0<t<25 min, increased k_{in} and k_{out} linearly during the (arbitrary selected) time interval 25min<t<50min, and kept k_{in} and k_{out} at their full nutrition value thereafter. Our predicted FIR as a function of time (Fig. S5) resembles the experimental observations of Fig. 6B.

S6. tRNA translocation in cells treated with puromycin

Fig. S6 provides enlarged images of the cells treated with puromycin (Fig. 7) in the presence (100%) and absence (0%) nutrition. The nuclear fluorescence intensity remains below the cytoplasmic fluorescence intensity both in the presence and absence of nutrition.

Barhoom et al. (2011, Fig. 2) observed that FIR increases about 2 fold when Chinese hamster ovary cells (CHO) transfected with Cy3-labeled tRNA are treated with puromycin under full nutrition conditions. We clearly have not observed such a pronounced effect under full nutrition, perhaps due to differences in the cell lines and internal rhd-tRNA concentrations achieved in the two experiments.

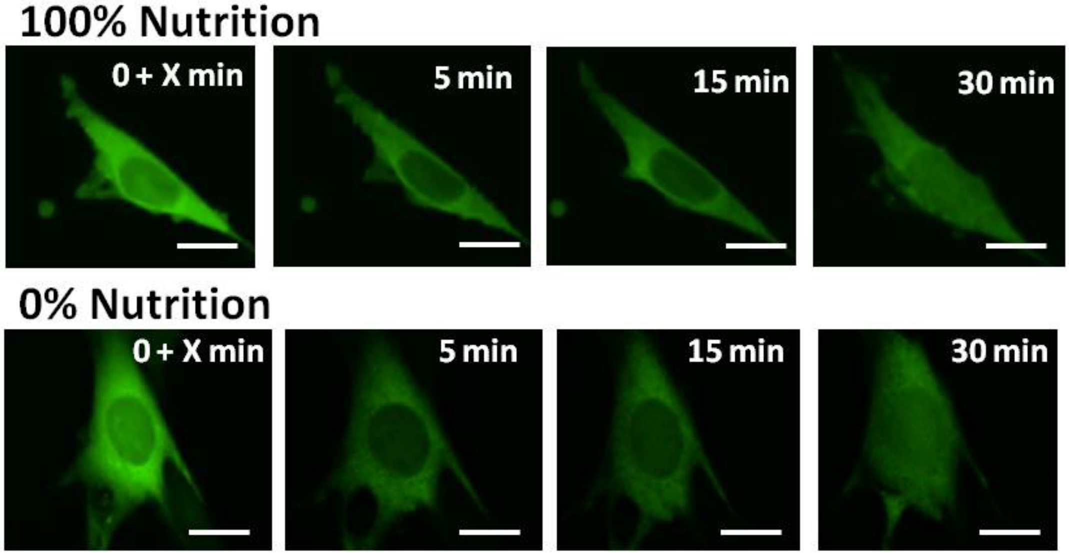

Fig. S6: Distribution of bulk tRNA co-injected with puromycin within 0.4 μm - thick confocal slices (located at MEF cells' mid-height) as a function of time under full (100%) nutrition (top raw) and no nutrition (bottom raw). Scale bar 25 μm. X < 15 seconds after tRNA+ puromycin injection into cytoplasm.

S7. Distribution of bulk rhd-tRNA in cells pre-exposed to 1extracellular microtubule de-polymerizing agent nocodazole under full (100%) nutrition.

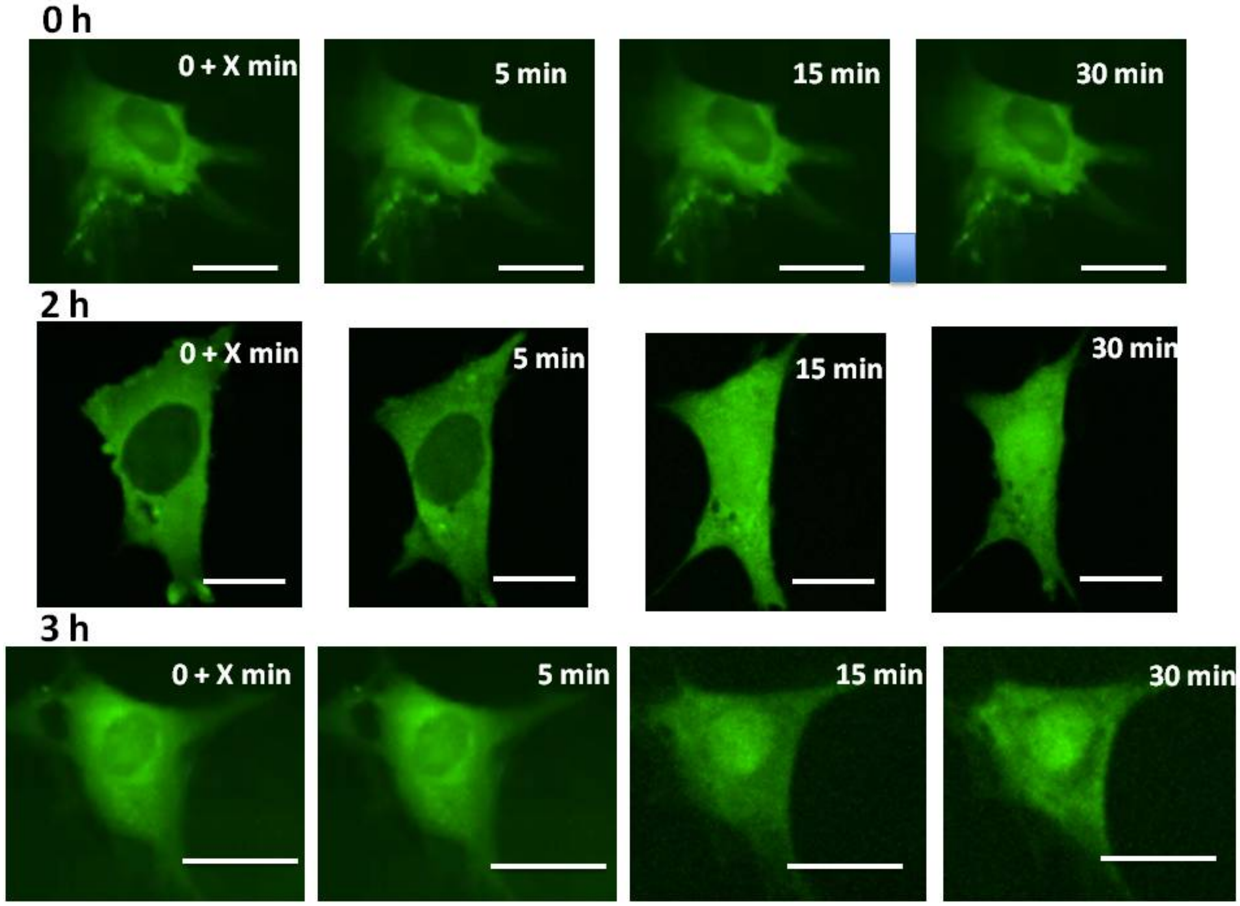

Fig. S7: Distribution of bulk rhd-tRNA at various times within 0.4 μm - thick confocal slices (located at MEF cells' mid-height) of cells pre-exposed for 0.02, 2, and 3 hours to 100nM extracellular microtubule de-polymerizing agent nocodazole under full (100%) nutrition. Scale bar 25 μm. Time zero corresponds to the injection time.

S8. Oligo distribution in the cytoplasm

To test whether specific binding of tRNA to organelles in the cytoplasm is responsible for the pattern of tRNA concentration distribution in the cytoplasm, we carried out a set of experiments with oligos. We hypothesize that the oligos are less likely to bind to cell components. We injected 25μM aqueous solution of fluorescently-labeled, 80bp long (similar in length to tRNA) oligonucleotides into the cytoplasm. The oligo sequence is: **5'-TAT AAG GCC TGC TGA AAA TGA CTG AAT ATA AAC TTG TGG TAG TTG GAG CTG ATG GCG TAG GCA AGA GTG CCT TGA CGA TA/36-FAM/-3'**.

S9. Micropipettes fabrication

A quartz glass capillary with filament (OD 1mm and ID 0.7mm, Shutter Instruments) was pulled with a pipette puller (Shutter Instruments P 2000) to produce two identical micropipettes of desired tip structure. We used a two step program with the heating rates and pulling velocities listed in **Table ST1**.

Table ST1: Puller Parameters

HEAT	FIL	VEL	DEL	PULL
850	5	40	145	60
750	5	50	135	180

The pulled pipettes were inspected with a microscope to ensure that they have the desired profile with a tip of diameter (<500 nm). **Fig. S8** shows two images of a pulled pipette at different magnifications.

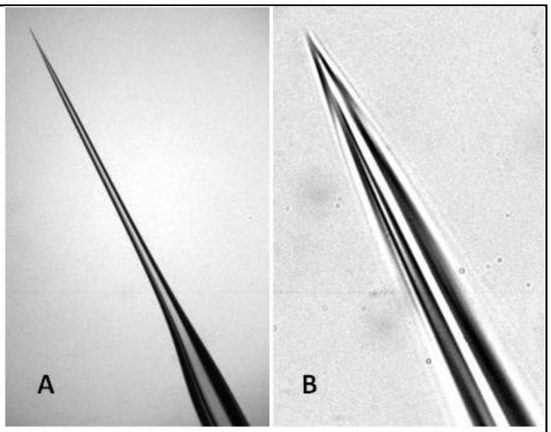

Fig. S8: Optical image of micropipette tips used for injecting tRNA (A) Low magnification image and (B) High magnification image of the tip. Scale bars (a) 0.5 *mm* and (b) 10 μm

S10. Supplement References

SR1. Alberts B., et al., 2007, Molecular Biology of the Cell. 6ed. Chapter 8. Taylor & Francis
